# Pediatric acute-onset neuropsychiatric syndromes and the gut-oral-brain axis: a narrative review of emerging microbiome-immune interactions and therapeutic perspectives

**DOI:** 10.3389/fimmu.2025.1726630

**Published:** 2025-11-27

**Authors:** Mariarosaria Matera, Valentina Biagioli, Maria Teresa Illiceto, Chiara Maria Palazzi, Ilaria Cavecchia, Andrea Manzi, Sebastian Lugli, Laura Pennazzi, Martina Meocci, Fausto Andrea Pedaci, Alexander Bertuccioli

**Affiliations:** 1 Microbiota International Clinical Society (MICS), Turin, Italy; 2 Department of Pediatric Emergencies, Misericordia Hospital, Grosseto, Italy; 3 Department of Neurosciences, Rehabilitation, Ophthalmology, Genetics, Maternal and Child Health, University of Genoa, Genoa, Italy; 4 Department of Pediatrics- Unit of Pediatric Gastroenterology and Endoscopy, Santo Spirito Hospital, Pescara, Italy; 5 Microbiomic Department, Koelliker Hospital, Turin, Italy; 6 Director of Pediatrics, Pozzuoli Hospital, Pozzuoli, Italy; 7 Ordinary Member of Microbiota International Clinical Society (MICS), Turin, Italy; 8 Italian Society of Nutraceuticals (SINut), Bologna, Italy; 9 Department of Respiratory Medicine, University of Siena, Siena, Italy; 10 Department of Pediatrics, Valdarno Hospital, Arezzo, Italy; 11 Department of Biomolecular Sciences, University of Urbino Carlo Bo, Urbino, Italy

**Keywords:** microbiota, immunology, interleukin-17, brain-gut axis, gastrointestinal microbiome, PANS, PANDAS, neuroinflammation

## Abstract

**Background:**

Pediatric Acute-onset Neuropsychiatric Syndromes (PANS) and Pediatric Autoimmune Neuropsychiatric Disorders Associated with Streptococcal infections (PANDAS) are characterized by sudden-onset neuropsychiatric symptoms. Growing evidence indicates that gut and oral microbiota may contribute to disease pathogenesis through immune and inflammatory pathways.

**Methods:**

This narrative review analyzed approximately 250 studies published between 2000 and 2024, retrieved from PubMed, Scopus, and Google Scholar. The selected works included clinical, immunological, and microbiome-related studies investigating the role of gut–oral–brain interactions in neuroinflammation or in pediatric PANS/PANDAS.

**Findings:**

Alterations in gut and oral microbial communities appear to modulate neuroinflammation through increased intestinal and blood–brain barrier permeability, immune dysregulation, and altered production of neuroactive metabolites. Specific bacterial families, such as *Bacteroidaceae*, *Rikenellaceae*, and *Odoribacteriaceae*, have been associated with pro-inflammatory states, while oral pathogens may exacerbate systemic inflammation via the gut–oral–brain axis.

**Conclusions:**

The reviewed evidence highlights the potential of microbiome-targeted strategies—including dietary modulation, probiotics, and anti-inflammatory approaches—as promising avenues for future personalized diagnosis and therapy in PANS/PANDAS. However, further controlled studies integrating microbial, immunological, and clinical data are required to confirm causal mechanisms and establish personalized therapeutic protocols.

## 1 Introduction

Acute-onset pediatric neuropsychiatric syndromes, such as PANS (Pediatric Acute-onset Neuropsychiatric Syndrome) and PANDAS (Pediatric Autoimmune Neuropsychiatric Disorders Associated with Streptococcal Infections), are heterogeneous neuroimmunopsychiatric conditions that result from multifactorial interactions between polygenic genetic factors and environmental influences (1).

They are characterized by a dramatic, sudden, or sometimes subacute onset of multiple neuropsychiatric symptoms in previously healthy children and adolescents. They may include: behavioral and cognitive oscillation and regression, obsessive-compulsive disorders (OCD), motor and vocal tics, anxiety, depression, sleep disorders (2, 3), urinary dysregulation symptoms such as overactive bladder, increased frequency and urgency of urination, enuresis (4), and food restriction (5). (**Tables 1** and **2**).

**Table 1:** PANDAS clinical criteria (2).

PANDAS clinical criteria	
1	TIC and/or OCD
2	Onset from 3 years to beginning puberty
3	Abrupt onset and relapsing/remitting course
4	Temporal relationship with GABHS infection
5	Association with neurologic abnormalities (hyperactivity, Tics or choreiform movements)

**Table 2:** PANS clinical criteria (3).

PANS clinical criteria	
1	Abrupt onset of OCD or Severe Restricted Food Intake
2	Association of al least two of the following neuropsychiatric symptoms: -anxiety -emotional lability and/or depression -irritability, aggression and/or oppositive behaviors -developmental and/or behavioral regression -deterioration in school performance -sensory or motor difficulty (hallucinations, altered sensitivity, dysgraphia, motor/vocal tics) -sleep disturbance -urinary disfunction (urinary frequency or enuresis)
3	No symptoms or sign of Sydenham chorea, systemic lupus erythematosus, Tourette syndrome or others neurologic disorder.
4	Association with *Mycoplasma pneumoniae*, *Borrelia burgdorferi*, *Staphylococcus aureus, Epstain-Barr*, Influenza HiN1, *Coxsackie virus*, Varicella-zoster virus, SARS-CoV-2 infections.
5	Association with environmental, toxic, metabolic or endocrine stress

They are also characterized by periods of remission and episodes of exacerbation during which a marked decline in performance is observed in several sensory domains, with a collapse in occupational performance, daily activity skills, and socialization, while persistent primary or secondary clinical progression is observed in some patients (6–8).

Although the clinical features of PANS and PANDAS have been increasingly characterized, the mechanisms linking peripheral infections to central neuroinflammation remain unclear. Emerging data point to the Oral-Gut-Brain Axis as a potential pathway mediating this connection. Alterations in the intestinal and oral microbiota may influence immune responses, neuroinflammatory cascades, and neurotransmitter regulation. Understanding how microbial dysbiosis contributes to acute neuropsychiatric symptom onset represents a crucial research gap. Focusing on this mechanistic hypothesis could provide new insight into disease pathogenesis and potential therapeutic targets.

### 1.1 Diagnosis

Since its first definition in 1998, PANDAS and PANS have been considered controversial diagnoses. To date, there are still a few conflicting studies and RCTs in PANDAS/PANS. As with many childhood neuroinflammatory diseases, diagnostic biomarkers and treatment protocols are lacking for these diseases (9).

Prato A et al., 2021 (10) conducted a systematic review of the literature to identify diagnostic criteria and procedures for PANS/PANDAS and proposed a panel of tests to support the physician for the diagnostic framing of these syndromes (**Table 3**). The authors emphasize that the diagnosis of these conditions is a clinical diagnosis of exclusion that requires a multidisciplinary approach.

**Table 3:** Diagnostic evaluations in PANS-PANDAS (10).

PANDAS	PANS
Cliincal evaluations	
Complete medical and psychiatric history	
Physical examination,	
Comprehensive neuropsychological assessment	
Laboratory test	
swab from the throat titers of antistreptolysin-O (ASO) and anti-DNase B.	possible triggering infectious agents (Mycoplasma pneumoniae, EBV, Borrelia burgdorferi, *Staphylococcus aureus*, Epstain-Barr virus, Influenza HiN1 virus, Coxsackie virus, Varicella-zoster virus, SARS-CoV-2 virus)10–13
complete blood cell count with manual differential	
indicator for liver and kidney disease	
inflammatory markers such us erythrocyte sedimentation rate (ESR) and C-reactive protein (CRP),	
metabolic panel	
urinalysis	
vitamin D levels	
Ferritin and iron levels	
thyroid function	
marker of thyroid autoimmune diseases	
marker of celiac disease	
IgG, IgA, IgM	
D8/D17 reactive cells monoclonal antibody directed against a B lymphocyte surface antigen	
antinuclear antibody (ANA), b2-glycoprotein antibodies, anticardiolipin antibodies, antiphospholipid antibody, and lupus anticoagulant	
autoantibodies to dopamine receptors D1 and D2, tubulin and lysoganglioside-GM1, and calcium/calmodulin dependent protein kinase II (CaMKII)	
anti-A carbohydrate (anti-ACHO) antibody	histone antibodies
	cytokine levels (IL1β, IL6, TNFα, and IL8, IL10)
Strumental tests	
Magnetic resonance images (MR), or Electroencephalography (EEG) if abnormalities complete with:	
complete cerebrospinal fluid (CSF) antineuronal antibodies	
Electrocardiogram and echocardiocolordoppler	
	polysomnography (PSG) in sleep disruptions

### 1.2 Treatments

Several treatment options have been proposed for PANS/PANDAS syndromes. However, the limited number of randomized controlled trials does not yet allow for the identification of a clear therapeutic protocol for the prevention and management of these neuropsychiatric diseases, in which immunomodulatory and anti-inflammatory approaches are used in association with psychotropic drugs and cognitive-behavioral psychotherapies.

Antibiotic therapy is useful in cases of active or persistent infection to eradicate the bacterial trigger and minimize the severity of PANDAS. Snider et al., 2005 have shown that neuropsychiatric exacerbations in these cases can be attenuated with the use of penicillin G, and azithromycin (11).

Sigra S et al., 2018 (12) conducted a systematic review of the literature related to the treatment of PANS/PANDAS with antibiotics, intravenous immunoglobulins (IVIG), therapeutic plasmapheresis (TPE), tonsillectomy, adenoidectomy, cognitive-behavioral therapy (CBT), nonsteroidal anti-inflammatory drugs (NSAIDS), corticosteroids, selective serotonin reuptake inhibitors (SSRIs), and neuroleptics. The authors noted that the studies are few, most are case reports, and in general have a moderate or high risk of bias, so they concluded that there is currently no concrete evidence to recommend a specific treatment in PANS/PANDAS.

Rea I. et al., 2021 (13) conducted a retrospective study on 62 PANS/PANDAS patients to evaluate the efficacy of antibiotic therapy combined with psychotherapy and antipsychotics in improving neurological symptoms and OCD. The multivariate analysis showed that psychotherapy produced a significant improvement in OCD (P 0.042) and that antibiotic prophylaxis improved acute neurological symptoms, but no significant efficacy was demonstrated in the long term.

Melamed et al., 2024 (14) investigated the role of IVIG administration in modulating inflammation and promoting resolution of infection in ten PANS patients (6–16 years), noting a reduction in myeloid pro-inflammatory cells (monocytes and dendritic cells) compared to pre-treatment levels with IVIG. Statistically significant improvements (p < 0.001) were also reported in all psychometric assessments, including the CY-BOCS scale (obsessive-compulsive scale), the YGTSS scale (tic scale).

Eremija et al., 2023 (15) also evaluated the efficacy of IVIG treatment through a retrospective study involving 12 children diagnosed with PANS. 11 out of 12 patients showed significant improvements in various neuropsychological domains after IVIG treatment, particularly in memory, sensory-motor skills, and visual-motor integration. Therefore, they concluded that immunomodulation via IVIG may represent an effective therapeutic option, especially in subjects with evidence of immune dysfunction such as hypogammaglobulinemia.

Han et al, 2025 (16) evaluated 31 PANS children among 100 total and reported that treatment with intravenous immunoglobulin (IVIg) in nine patients partially normalized the altered pathways: ribosomal and epigenetic processes shifted toward a profile closer to controls. Clinically, IVIg improved obsessive symptoms, but the effects were temporary and waned before the next infusion.

Latimer ME, et al., 2015 (17) conducted a retrospective review involving 35 pediatric PANDAS patients treated with TPE at Georgetown University Hospital, between August 2009 and October 2013, noting improvements in OCD, anxiety, tics, and somatic symptoms such as dysgraphia, sleep disturbances, and urinary urgency or frequency, with an average improvement of 65% at 6 months after TPE and 78% at a long-term follow-up. They concluded that TPE is an invasive, but safe, well-tolerated and beneficial medical intervention that should be reserved for children and adolescents with severe symptoms of PANDAS.

According to the 2019 American Society for Apheresis (ASFA) guidelines, the use of TPE, aimed at removing autoantibodies from plasma, is an invasive medical intervention with a second-line therapeutic indication in PANDAS patients with exacerbation (18). In addition, Prus K et al., 2022 (19) evaluated for the first time the response to TPE even in adult PANDAS/PANS patients with a late stage and prolonged symptomatic history, detecting clinical improvement in more than half of the patients.

Johnson M, et al., 2021 (20) carried out a systematic literature search (from 1998 to June 2020) assessing the effects of treatment in PANS patients (<18 years). They evaluated the use, versus non-use, of anti-inflammatory, antibacterial, or immunomodulatory treatments. Two RCTs investigated antibiotics (penicillin V, azithromycin) and one RCT investigated immunomodulatory treatments (IVIG and TPE). Regarding symptoms, two non-RCT studies evaluated anti-inflammatory treatment (cyclooxygenase inhibitors (COX), corticosteroids), two RCTs and one non-RCT evaluated antibiotics (penicillin G, azithromycin), and two RCTs evaluated immunomodulatory treatments (IVIG, TPE). The results of this systematic review, based on the currently available evidence, indicate that there is moderate certainty that such treatments may result in adverse effects and that their efficacy in the treatment of hypothesized neuroinflammation or autoimmunity is uncertain or potentially limited.

Cocuzza S et al., 2022 (21) conducted a systematic literature review with meta-analyses over the past 20 years to 2022, to investigate medical and surgical treatment strategies. The authors examined 11 studies involving a total of 473 patients, of which four included 129 surgical subjects and seven investigated 326 pharmacologically treated patients. The analysis of the pooled results showed that surgical and pharmacological treatment reported a reduction in OCD, but no statistical significance was obtained (p < 0.05 for both). The authors therefore concluded that surgical therapy in selected patients may lead to promising results, although more evidence is needed.

Up to now, the role of medical therapy (**Table 4**) remains controversial, often due to the lack of randomized clinical trials, comparable therapeutic protocols, and variable responses depending on the drug used and the time of administration.

**Table 4:** Therapeutic approaches currently in use in PANS-PANDAS syndromes.

Treatments in use in PANS/PANDAS
antibiotics
nonsteroidal anti-inflammatory drugs (NSAIDS)
Corticosteroids
selective serotonin reuptake inhibitors (SSRIs)
neuroleptics
immunoglobuline endovenose (IVIG)
therapeutic plasmapheresis (TPE)
tonsillectomy
adenoidectomy

### 1.3 The role of microbiomics

Studies so far, conducted in animal and human models, and neuroimaging studies, have shown how infectious and inflammatory triggers can activate abnormal immune responses and compromise blood-brain barrier (BBB) function, allowing antibodies and mononuclear cells to access the central nervous system (CNS), especially at the level of the basal ganglia, confirming how neuroinflammation plays a crucial role in the pathogenesis of these conditions.

Recently, microbiomics, i.e., the study of the gut microbiota, has emerged as a promising field for its potential role in neuropsychiatric diseases and in the diagnosis and management of acute-onset pediatric neuropsychiatric syndromes. Alterations in the gut microbiota can affect barrier permeability, functional homeostasis of the immune system, and contribute to the predisposition to develop neuroimmune conditions such as PANS/PANDAS. Therefore, microbiota modulation could represent a new therapeutic strategy in these patients (9).

In this review, we analyze the latest scientific research on the potential role and clinical applications of microbiomics in PANS-PANDAS.

## 2 Materials and methods

### 2.1 Search strategy

A comprehensive literature search was conducted to identify peer-reviewed articles exploring the clinical and pathophysiological aspects of PANS and PANDAS, with particular focus on immune dysregulation, neuroinflammation, and Scopus, search terms included combinations of “PANS”, “PANDAS”, “microbiota”, “gut-brain axis”, “oral microbiota”, “neuroinflammation”, “IL-17”, “autoantibodies” and “streptococcal infection”. The search was limited to articles published between 2020 and 2025, and preference was given to original research, systematic reviews, and meta-analyses published in English.

### 2.2 Inclusion and exclusion criteria

Inclusion criteria were studies involving pediatric subjects diagnosed with PANS or PANDAS; studies focusing on immunological, neuroinflammatory, or microbiome-related mechanisms; clinical and preclinical studies providing original data or validated analysis, systematic reviews, and meta-analyses relevant to the scope of PANS-PANDAS and microbiome interaction. Exclusion criteria were studies limited to adult populations, articles lacking relevance to the gut-brain-immune axis, papers without rigorous methodology, and non-English language publications without an available translation.

The review considered a wide array of study designs to ensure a multidisciplinary perspective such systematic reviews and meta-analyses that synthesized data on diagnosis and treatment, randomized controlled trials (RCTs), and observational studies evaluating immunomodulatory or antimicrobial interventions, retrospective cohort studies and clinical case series documenting therapeutic outcomes in real-world settings, preclinical studies, particularly involving germ-free models and microbiota transfer, elucidating causal links between microbiota alterations and neuroinflammation, microbiome sequencing studies (e.g., 16S rRNA metagenomics) identifying potential diagnostic or prognostic microbial biomarkers in the gut and oral cavity.

## 3 Pathophysiology and immune mechanisms

### 3.1 Role of infections and autoimmunity

Although it is challenging to establish a direct causal relationship, it is believed that PANS/PANDAS may manifest in genetically predisposed individuals following exposure to infectious stimuli. Trifiletti et al., 2022 (1) conducted a whole-exome sequencing analysis on 386 patients with PANS and a whole-genome sequencing analysis on 10 patients with severe PANS; *de novo* or ultra-rare mutations were found in 21 patients affecting 11 genes involved in the peripheral regulation of immune responses, microglia, and neuronal synaptic function.

On such a genetic substrate, an infectious trigger could be decisive in activating a persistent dysfunction of the immune system. In particular, PANDAS recognizes group A β-hemolytic Streptococcus (GABHS) (2, 22), as a specific trigger. At the same time, PANS seem to begin or show exacerbation following bacterial infections (*Mycoplasma pneumoniae, Borrelia burgdorferi, Staphylococus aureus*), viral infections (*EpstEin-Barr, Influenza* H1N1, *Coxsackie virus, Varicella-zoster virus*, SARS-CoV-2) (10, 23–25) or sometimes following exposure to non-infectious triggers (environmental, toxic, endocrine and metabolic), capable of abnormally activating the immune system, exacerbating an abnormal inflammatory response of the central nervous system (25).

Currently, the pathophysiological mechanisms of PANS/PANDAS are not yet fully understood; however, an autoimmune immune-mediated etiology is suspected for both PANS and PANDAS (26). In support of this thesis, biomarkers typically related to Sydenham chorea, such as autoantibodies against dopamine receptors (DR) DR1 and DR2, lysoganglioside-GM1, and β-tubulin, have also been detected in PANS, as previously proposed with the Cunningham panel for PANDAS (27). Cunningham’s panel includes antibodies directed against D1R, D2R, lysoganglioside-GM1, and tubulin and calcium-calmodulin-dependent protein kinase (CaMKII) activity; however, it should be emphasized that these panels need further validation (28–30).

The CNS is supposed to be attacked by an aberrant immune response and persistently activated by an infectious trigger or other environmental factors. Immunological activation would persist even after the remission of the acute phase, causing damage and functional alteration of the CNS (31, 32). Chain et al. (33) reported elevated levels of antineuronal autoantibodies and elevated calcium/calmodulin-dependent kinase II (CaMKII) activity in both serum and cerebrospinal fluid from 32 patients with acute symptomatic PANDAS. Interestingly, although some healthy controls had elevated levels of antibodies, none showed a significant increase in CaMKII activity, suggesting that CaMKII could play a significant role in the neuroinflammatory process.

Immunological changes have been reported both during and after exposure to environmental triggers. In addition, elevated levels of pro-inflammatory cytokines and autoantibodies capable of crossing the compromised BBB have been observed, cross-binding with brain proteins of neuronal and non-neuronal cells, and with neurotransmitter receptors, mainly within the basal ganglia (34). Post-infectious neuroinflammation has been studied in animal models using human sera, and these studies have confirmed that autoantibodies, by cross-reacting with neural antigens in the basal ganglia, modulate neuronal activity (35, 36).

Wells et al., 2024 (37), in a study involving PANS and PANDAS patients, showed that 63.8% of patients have autoantibodies against the α-folic acid (FR) receptorα. FRsα are associated with both brain folate deficiency (CFD) and autism spectrum disorders (ASD), both syndromes with features that sometimes overlap with PANS/PANDAS. Therefore, the authors suggest that FRsα could contribute to PANS/PANDAS symptomatology and that leucovorin administration could result in improvement of symptoms in FR-positive patients.

In a recent study in murine model White et al. (38) underlined that T cells that recognize gut-colonizing segmented filamentous bacteria can drive inflammation in both the intestine and the CNS when functional regulatory T cells are absent. Dysregulated gut commensal–specific CD4 T cells (Tcomm cells) in the inflamed intestine may acquire the ability to infiltrate the CNS regardless of their original antigen specificity. Once inside, they can be reactivated by host-derived protein antigens through molecular mimicry, leading to the production of high levels of GM-CSF, IFNγ, and IL-17A, which trigger neurological damage.

Within the CNS, infiltrating Tcomm cells initiate inflammation by activating microglia through two complementary mechanisms: an IL-23R–dependent encephalitogenic program and IL-23R–independent GM-CSF production. Together, these findings highlight potential pathways by which perturbations in Tcomm cells contribute to extraintestinal inflammation.

Recently Han et al. (16) propose that PANS is not a classic autoimmune disease but rather a neurodevelopmental phenotype driven by epigenetic dysfunction, disrupting translational and immune processes. They suggest that early-life environmental factors modify the epigenome and epitranscriptome of immune cells, influencing brain development through a “two-hit” mechanism (genetic vulnerability + infection/stress). The study included 100 children: 32 with PANS, 68 with other NeuroDevelopmental Disorders, and 58 neurotypical controls. Findings showed that PANS patients had more early-life infections and loss of acquired skills compared to controls. Although routine immune testing appeared normal, RNA sequencing revealed profound alterations: upregulation of genes related to ribosomal biogenesis and RNA methyltransferases, downregulation of cellular functions such as mitochondrial activity, signaling, endocytosis, and immune responses. Single-cell RNA-seq confirmed these findings, highlighting heterogeneity across immune cell types. The Toll-like receptor (TLR) stimulation assay showed reduced inflammatory responses (TNF-alpha and IL-6) in PANS patients, indicating innate immune dysfunction. While the multi-omic approach and integration of transcriptomic and functional data strengthen the study’s biological plausibility, its cross-sectional design and moderate sample size limit causal inference. Moreover, the absence of longitudinal follow-up and potential confounders (e.g., comorbid NDDs, medication exposure) may have influenced gene expression profiles. As such, the level of evidence can be considered moderate, suggesting a compelling mechanistic hypothesis rather than definitive proof of causality.

### 3.2 IL-17 and Th17 response

Interleukin-17 (IL-17) is a pro-inflammatory cytokine secreted mainly by T helper 17 (Th17) cells and has recently drawn considerable attention for its contribution to autoimmune and neuroinflammatory diseases. Among the IL-17 cytokine family (IL-17A–E) (39), IL-17A is the best characterized effector molecule, binding to the IL-17RA receptor and activating intracellular pathways such as MAPK (mitogen-activated protein kinase) and NF-κB (nuclear factor Kappa-light chain enhancer of activated B cells). Through these signaling cascades, IL-17 regulates the expression of pro-inflammatory cytokines (IL-6, TNF-α, IL-1β), chemokines, and matrix metalloproteinases, amplifying the local inflammatory response and sustaining tissue injury (40, 41).

Under physiological conditions, IL-17 plays a protective role by recruiting neutrophils and stimulating the production of antimicrobial peptides (AMPs), thereby promoting host defense and tissue repair (42). However, under chronic inflammatory conditions, sustained IL-17 signaling perpetuates autoimmunity and neuroinflammation. Th17 cells can also secrete IL-21, IL-22, GM-CSF, and IFN-γ; their inflammatory profile depends on the surrounding cytokine milieu. Normally, Th17 cells co-express IL-10, maintaining an equilibrium between pro- and anti-inflammatory responses. When this balance is lost, excessive IL-17, IL-22, and GM-CSF release establishes a self-perpetuating inflammatory loop (42–44).

Experimental studies have demonstrated several mechanisms by which IL-17 contributes to neuroinflammation.

**Figure 1:**
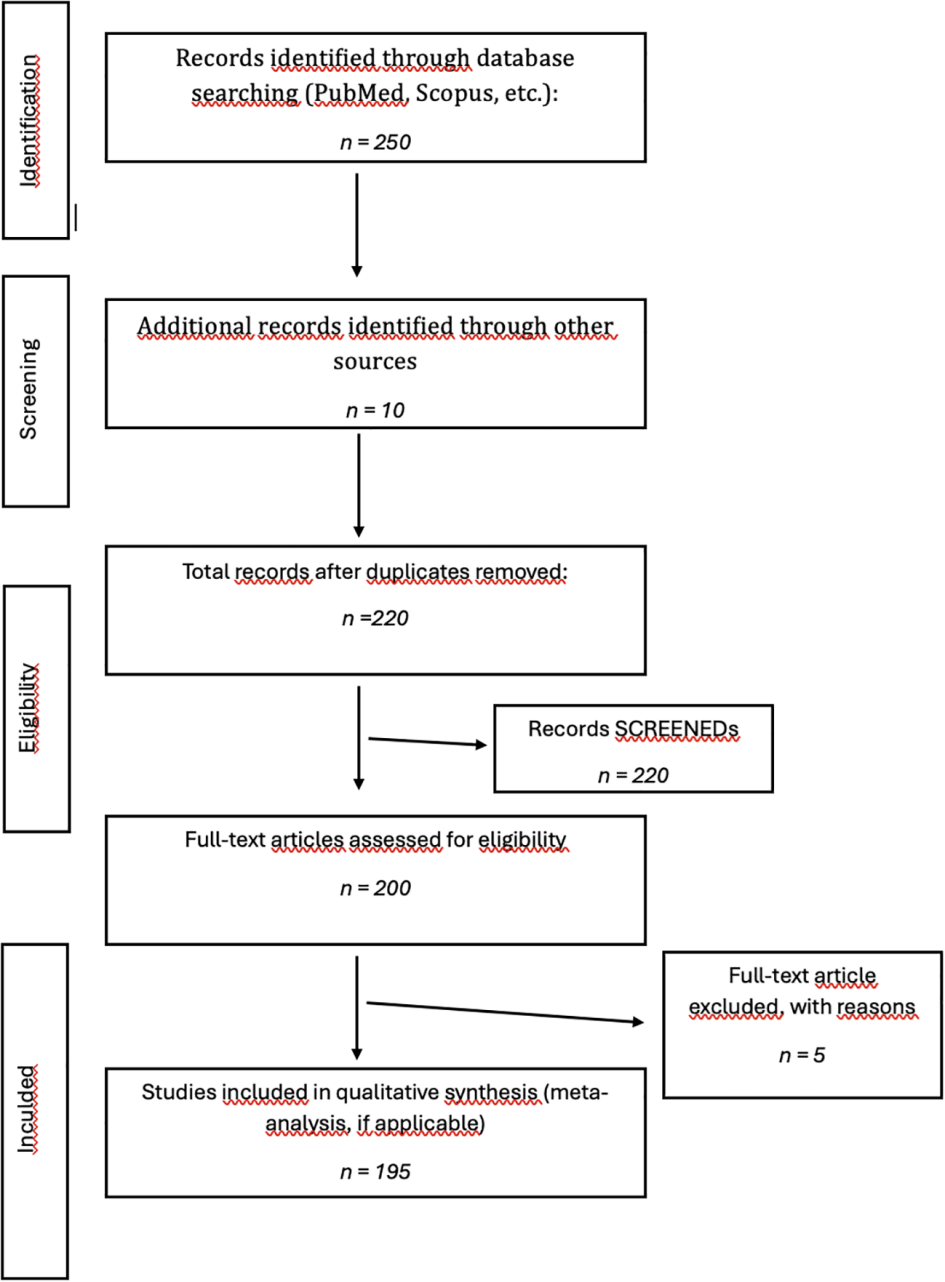
Flow-chart PRISMA.

- Disruption of the BBB. Numerous studies conducted on the animal model of multiple sclerosis (MS), such as experimental autoimmune encephalomyelitis (EAE) mice, have revealed that IL-17 induces damage to the BBB. Dileepan T et al, 2016 (45) studied a mouse model of post-infectious BGE (basal ganglia encephalitis) developed through intra-nasal infection with live Streptococcus. In this model, Streptococcus-specific Th17 cells proliferate and differentiate into nasal lymphoid tissue, from which they then migrate, via the olfactory nerve path, to the CNS, resulting in increased BBB permeability and microglial inflammatory activation. Subsequently, the same group (46) confirmed that it is precisely Th17 cells, altering the permeability of the BBB, that allow IgG to enter the CNS, since mice genetically lacking Th17 lymphocytes, subjected to similar infectious stimulation, had less damage to the BBB, less antibody infiltration into the CNS, and less microglial inflammatory activation. Once the integrity of the BBB is compromised, there is a greater influx of Th17 cells, neutrophils, or δβ-T cells within the brain parenchyma, with a further increase in IL-17 production and the initiation of dysfunction, first and then irreversible neuronal damage (44, 47).
- Impaired cell differentiation. Several studies conducted on animal models have shown that IL-17 influences the proliferation and differentiation of neural stem cells and neural progenitor cells involved in neurogenesis processes, reduces the proliferation of hippocampal neural progenitor cells in the early stages of neurogenesis, and promotes neuronal differentiation and maturation in the later stages (48, 49).
- Microglial activation. IL-17 drives microglia toward a pro-inflammatory M1 phenotype, leading to excessive cytokine release and neuronal apoptosis (50, 51). Conversely, IL-17 blockade (i.e. the use of anti-IL-17 antibodies) promotes an M2 anti-inflammatory, neuroprotective profile and restores cognitive performance (52). In addition, the microglia themselves, when activated in an inflammatory M1 sense, release IL-17, which promotes neuronal apoptosis, creating a vicious circle of inflammatory self-maintenance (53).
- Altered neurotransmission. IL-17 interferes with GABAergic signaling (54), reducing inhibitory tone and contributing to mood and cognitive disturbances observed in neuropsychiatric conditions (55, 56).
- Microbiota interaction. IL-17 and the gut microbiota are tightly linked. Studies conducted on mice lacking IL-17R have also confirmed the existence of a delicate balance between IL-17 and microbiota: IL-17 regulates bacterial growth and intestinal ecology, which in turn and first of all segmented filamentous bacteria (SFB) (57), stimulates the activation of Th17 cells (58–60) with a consequent increase in IL-17 secretion reinforcing a bidirectional gut–immune–brain feedback loop (61, 62) (**Figure 1**).

### 3.3 The IL-17 axis in neuroinflammation

**Figure 2:**
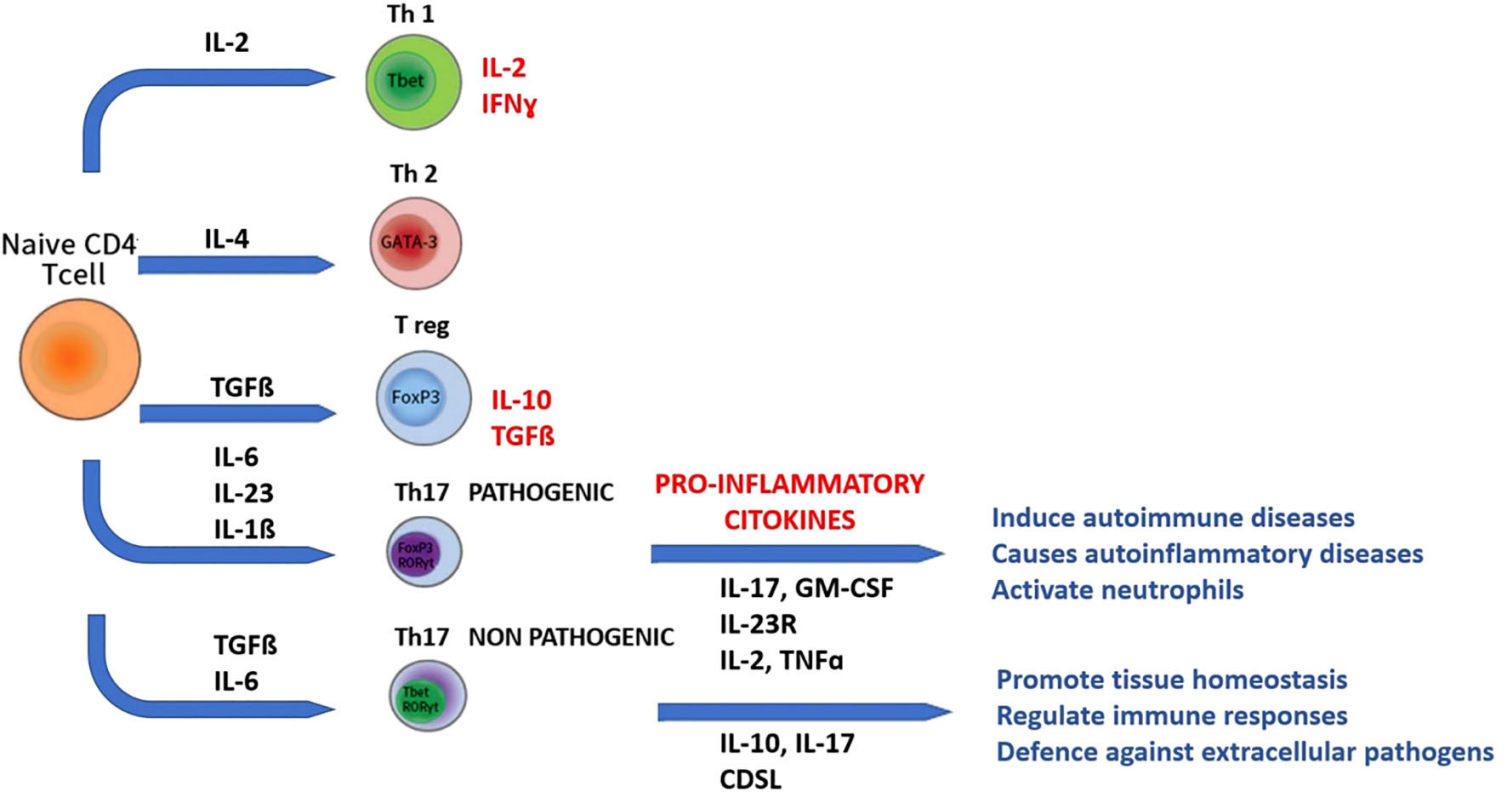
The IL-17 Axis in Neuroinflammation: cytokine network regulating Th17 differentiation and IL-17 production from naïve CD4^+^ T cells. The figure summarizes the main cytokines (IL-6, IL-1β, IL-23, TGF-β) and transcription factors (STAT3, RORγt) involved in Th17 differentiation, and the effector cytokines (IL-17A, IL-21, IL-22, GM-CSF) contributing to neuroinflammation in PANS/PANDAS.

Th17 cells represent a new lineage distinct from Th1 and Th2 cells. In murine models, recently a new lineage of CD4+T cells, Th17 cells producing the signature cytokines IL-17, IL-21, and IL-22, has been identified. This has fundamentally changed the Th1/Th2 dichotomy paradigm (**Figure 2**). The Th1 cell subset is characterized by IFN-γ production and T-bet expression, whereas the Th2 cell lineage shows IL-4, IL-5, and IL-10 production and GATA-3 expression. At the other extreme, Treg cells expressing FoxP3 are essential to maintain the homeostasis of cell subsets involved in adaptive immunity by contact-dependent suppression or by releasing anti-inflammatory cytokines, IL-10, and TGF-β1. Like Th1, Th2, and Treg cells, specific polarizing cytokines, TGF-β, IL-6, and IL-23, are required for differentiation of Th17 cells, which express the transcription factors retinoic acid orphan receptor (ROR)α and RORγt. However, Th17 cells do not represent a homogeneous lineage, as they can be reprogrammed to other T-cell subsets depending on the cytokine environment. Accordingly, IL-17 can be co-expressed with a variety of other cytokines, including IFN-γ, IL-10, and IL-4. Indeed, a mixed Th1-Th17 subgroup expressing both T-bet and RORγt has been recently identified in the setting of autoimmunity. Plasticity between Th17 and Treg cells has also been observed. In humans, IL-17-producing ROR γt+FoxP3+T cells that retain their ability to suppress effector lymphocytes have been described in **Figure 2**. Because of these recent acquisitions, it is now clear that Th17 cells produce cytokines that mediate immune responses that are both good and bad.

Th17 cells have been shown to have either a pathogenic or protective role in diseases such as EAE or autoimmune uveitis (EAU). Through their expression of the transcription factor RORγt, Th17 cells play critical roles in the development of autoimmunity and allergic reactions by producing IL-17 and, to a lesser extent, TNF-α and IL-6 (63).

Foiadelli T. et al., 2025 (64) analyzed serum and cerebrospinal fluid (CSF) concentrations of IL-17 in a cohort of 58 patients with acute neuropsychiatric disorders (50.8% classified as PANDAS and 11.8% as PANS). Mean serum IL-17 concentrations were significantly higher in the study group than in controls (p < 0.0001), so the authors concluded that IL-17 could be involved in the pathogenesis of acute neuropsychiatric disorders in childhood. In fact, it is hypothesized that the action of T cells, in particular Th17 lymphocytes, is involved in the triggering of neurovascular dysfunction through BBB damage (65).

When separately considering the final diagnosis, IL-17 was significantly higher in CSF than in the serum of PANDAS patients, but this result was not confirmed by multivariate mixed model analysis.

They noted that the IL-17 level did not decrease with time, and the analysis of their data suggests that correlation between higher serum and CSF-IL17 concentration and later sampling (> 12 months) from the onset of neuropsychiatric symptoms.

Moreover, microbial and streptococcal infection–induced Th17 expansion facilitates CNS infiltration and IL-17 release within the basal ganglia, where it alters neurotransmission and triggers PANDAS-like behaviors such as tics and obsessive–compulsive symptoms. These findings reinforce the hypothesis that the microbiota-modulated Th17/IL-17 response represents a critical immunological link between infection, barrier dysfunction, and neuroinflammation in PANS/PANDAS.

Finally, the reciprocal relationship between Th17 and regulatory T (Treg) cells is crucial for maintaining immune tolerance. A shift toward a higher Th17/Treg ratio has been consistently observed in inflammatory neurological diseases and may represent a key immunopathological feature of PANS/PANDAS. Restoring this balance could therefore emerge as a potential therapeutic target.

### 3.4 Neuroinflammation, barrier dysfunction, and the gut–brain axis

The maintenance of CNS homeostasis relies on the integrity and selective permeability of vascular barriers, namely the BBB, the gut vascular barrier (GVB), and the plexus vascular barrier (PVB). While the BBB and GVB are highly restrictive, the PVB exhibits greater permeability due to fenestrations regulated by Plasmalemmal Vesicle-associated Protein-1 (PV-1), whose expression is modulated by inflammatory stimuli (66, 67).

Under physiological conditions, a functional GVB ensures that only beneficial metabolites enter the bloodstream, maintaining a “safe” circulation for the CNS and allowing the PVB to remain open to support molecular exchange essential for neuronal function (66).

Conversely, in intestinal dysbiosis and inflammation, GVB integrity deteriorates, permitting translocation of bacteria and endotoxins into the systemic circulation. In response, the PVB reduces its permeability to prevent the entry of harmful molecules into the CNS; however, this non-selective restriction also impedes nutrient transport and waste clearance, promoting neuroinflammation and neuronal dysfunction. This dynamic illustrates the critical role of the GVB–PVB axisin linking metabolic and inflammatory disturbances to CNS pathology, as observed in disorders such as PANS/PANDAS and neurodegenerative diseases (68, 69).

The gut microbiota exerts a pivotal influence on this axis through its role in regulating intestinal barrier function and systemic immunity. In eubiotic conditions, commensal microbes contribute to barrier integrity via the production of short-chain fatty acids (SCFAs)—notably acetate, propionate, and butyrate—which provide energy to colonocytes, enhance tight junction stability, and stimulate antimicrobial peptide synthesis (70–73). Butyrate also acts epigenetically through inhibition of histone deacetylases (HDACs), promoting the expression of anti-inflammatory mediators (e.g., IL-10, TGF-β) and supporting the expansion of regulatory T cells (Tregs) (74–77).

Beyond the gut, SCFAs influence CNS homeostasis by strengthening BBB integrity and modulating neuroimmune signaling. They upregulate tight junction proteins (occludin, claudins, ZO-1) through activation of Nrf2 and Wnt pathways and by suppressing NF-κB–mediated inflammatory responses (78–84).

In microglia, SCFAs promote a shift toward an anti-inflammatory phenotype and reduce pro-inflammatory cytokine production (85–90). Experimental evidence indicates that dietary fiber deficiency, which reduces SCFA levels, leads to BBB disruption, microglial activation, synaptic loss, and cognitive impairment—effects reversible upon SCFA supplementation (91).

Conversely, dysbiosis-associated increases in intestinal permeability facilitate the translocation of lipopolysaccharides (LPS) from gram-negative bacteria such as *Proteobacteria* and *Bacteroides* into the bloodstream, a condition termed metabolic endotoxemia (92, 93). Circulating LPS activates Toll-like receptors (TLR2/4) in macrophages and microglia, inducing chronic systemic and neuroinflammation implicated in metabolic, autoimmune, and neuropsychiatric disorders (94–101).

Overall, the gut–brain axis represents a finely tuned network linking microbial metabolism, vascular barrier integrity, and neuroimmune regulation. Disruption of this equilibrium—through dysbiosis, barrier dysfunction, or inflammatory signaling—compromises BBB function and promotes persistent neuroinflammation, providing a mechanistic framework connecting gut-derived inflammation to CNS disorders.

## 4 Gut microbiota and neuroimmune interactions

### 4.1 SCFAs, neurotransmitters, and microglial regulation

Neurotransmitter biosynthesis is another possible way in which the microbiota may influence brain function. For example, gut microbiota, through the production of tryptophan, regulate serotonin biosynthesis by enterochromaffin cells of the gastrointestinal tract. *Actinobacteria* (i.e., *Bifidobacterium longum*), *Firmicutes* (i.e., Lactic acid bacteria like the genus *Lactobacillus*), *Bacteroidetes*, *Proteobacteria*, and *Fusobacteria* are the main bacterial phyla responsible for producing and metabolizing tryptophan in the gut (102). Bacterial output (i.e, SCFA) can also influence serotonin synthesis. For example, one study found that acetate and butyrate promoted tryptophan hydroxylase 1 (the rate-limiting enzyme for mucosal serotonin synthesis) transcription in a human-derived enterochromaffin cell model, suggesting that SCFAs might be crucially involved in enteric serotonin production and homeostasis (103). Importantly, the pro-inflammatory environment in the intestine due to gut dysbiosis can shift the production of serotonin to the kynurenine pathway the result in reduced peripheral and central serotonin levels. Mechanistically, dysbiosis leads to increased gut permeability and elevated systemic and mucosal pro-inflammatory cytokines (e.g., TNF-α, IL-6, IFN-γ), which upregulate indoleamine 2,3-dioxygenase (IDO) and tryptophan 2,3-dioxygenase (TDO) activity (104). These enzymes shunt tryptophan metabolism toward kynurenine and its metabolites, reducing substrate availability for serotonin biosynthesis in enterochromaffin cells and also contributing to neuroinflammation and altered gut-brain axis signaling. Indeed, dysbiosis and inflammation downregulate the expression and activity of kynurenine aminotransferases (KATs), the enzymes responsible for converting kynurenine to kynurenic acid, favoring the production of other neurotoxic metabolites like quinolinic acid. Kynurenic acid has anti-inflammatory and neuroprotective properties, while quinolinic acid favors neuroinflammation because it activates N Methyl D Aspartate NMDA receptors and promotes excitotoxicity and microglial activation in the CNS (105, 106).

Some gut bacteria are also important producers of gamma-aminobutyric acid (GABA) (107). The main GABA-producing bacterial species in the gut are *Bifidobacteria* (i.e., *B. adolescentis*), *Lactobacillus* spp. (notably *L. plantarum* and *L. brevis*), *Bacteroides* spp. (i.e., *B. fragilis*), and *Akkermansia muciniphila*. Gut dysbiosis associated with the reduction of key GABA-producing taxa (especially *Bifidobacterium* and *Lactobacillus*) and with a pro-inflammatory gut environment can impair GABAergic homeostasis in the gut and brain. Indeed, inflammation-driven disruption of the gut barrier increases permeability, allowing translocation of microbial products (i.e, LPS) and pro-inflammatory cytokines (i.e., TNF-α, IL-1β) into the circulation. These mediators reach the brain, activate microglia, and promote neuroinflammation. Cytokines and microbial products also downregulate the expression and function of GABA _A_ and GABA _B_ receptors and GABA transporters in both the gut and brain, as demonstrated by altered transcriptomic profiles and receptor subunit expression in dysbiotic states (107). The disruption of GABAergic signaling leads to reduced inhibitory tone, increased neuronal excitability, and impaired gut-brain axis homeostasis, thereby amplifying neuroinflammation.

Another important pathway regulated by gut microbiota is the dopamine (DA) pathway. DA is the main catecholamine neurotransmitter in the mammalian CNS. Alterations in dopaminergic transmission have been associated with CNS disorders, such as anxiety, attention deficit hyperactivity disorder (ADHD), Parkinson’s disease, and compulsive food intake, among many others (108). The synthesis of DA occurs through the phenylalanine-tyrosine-dopa-dopamine pathway, which can take place both in the gut and brain. For example, *Clostridium* (i.e. *C.* sp*orogenes*) and *Bifidobacterium* (i.e., *B. dentium*) are shown to produce phenylalanine (the former) and tyrosine from phenylalanine (the latter) (109, 110). Furthermore, gut microbiota can affect host DA transporters (DAT), a protein responsible for DA reuptake and clearance of synaptic space in the brain. Studies have shown that an increase in fecal levels of *Bacteroides uniformis* is significantly correlated with an increase in DAT, while an increase in the *Prevotella* genus is negatively correlated (111).

Increased DAT leads to enhanced reuptake of dopamine from the synaptic cleft into presynaptic neurons, thereby reducing extracellular dopamine availability and synaptic signaling. This reduction in dopaminergic tone is implicated in neuropsychiatric disorders such as depression, ADHD, and Parkinson’s disease, where low synaptic dopamine is a hallmark (112). Evidence that the intestinal microbiota is important in modulating the dopaminergic pathway also comes from studies on rodents lacking intestinal microbes (i.e., germ-free animals) in which we observe a dysregulation of dopaminergic signaling with an excess of DA receptor expression, DA levels, and DA turnover (108).

### 4.2 Microbial signatures

Microorganisms have always been an essential component of human life. Coevolution with humans has allowed for a mutualistic and symbiotic development in which the host provides ecological niches where microorganisms can live and proliferate within an optimal microenvironment (with a certain pH, oxygen, humidity, and nutrient availability), and the microbes, in return, provide the host with their genomic potential for synthesizing molecules essential to the individual’s health. The gastrointestinal (GI) tract is populated by bacteria, fungi, viruses, and archaea concentrated in the colon, and it is estimated that this community, called the gut microbiota, contains 150 times more genes than the entire human genome (113).

This genetic diversity also consists of diverse metabolic and bioactive potentials; for this reason, preserving microbial biodiversity is essential for maintaining what is known as eubiosis.

Eubiosis refers to a balanced state of the microbiota capable of maintaining functional homeostasis not only locally, at the intestinal level, but also promoting the health of the host organism. Dysbiosis, on the other hand, is the disruption of this balance, resulting in both quantitative and qualitative alterations of the bacterial ecosystem, altered barrier permeability, and the translocation of inflammatory molecules, microbes, bacterial metabolites, and immune cells across the GVB and central barriers such as the BBB and PVB, resulting in systemic inflammation and neuroinflammation (66, 114).

The intestinal microbiota can communicate even with seemingly distant organs; for this reason, we speak of gut-organ axis. Therefore, the microbiota-gut-brain axis (MGBA) is undoubtedly the most studied due to its increasingly evident correlation with neurodegenerative and neurodevelopmental diseases. Accumulating evidence has highlighted a bidirectional communication between the gut microbiota and the CNS. Various mechanisms have been proposed that contribute to this reciprocal communication; the key players are the intestine, complex system of peripheral barriers (primarily the intestinal one, and central barriers such as the BBB and PVB), the enteric nervous system (ENS), vagus nerve, the immune system, the circulatory system, neuroendocrine system with the hypothalamic-pituitary-adrenal (HPA) axis. Moreover, it has been demonstrated that the gut microbiota and specific bacteria can produce metabolites, such as SCFAs including butyrate, acetate, and propionate, as well as hormones and neurotransmitters such as melatonin, dopamine, serotonin, and GABA. They are also capable of synthesizing vitamins (such as vitamins B and K) (115). Studies in animal models have demonstrated how a dysbiotic microbiota can be an important driver of neuroinflammation mediated by modulating microglial activation.

Microglia are the primary immune cells of the CNS and can present in different phenotypic (ramified and amoeboid) and functional forms (pro-inflammatory M1 and anti-inflammatory M2). In homeostatic conditions, microglia perform the task of monitoring the brain environment, guiding neurogenesis, promoting synaptic transmission, myelin health, and maintaining BBB integrity. However, in pathological neuroinflammatory conditions, they lose their functional balance and the ability to transition from one phenotypic and functional state to another.

In chronic neuroinflammatory conditions, microglia remain persistently activated in a pro-inflammatory M1 mode, releasing inflammatory chemokines and cytokines that support neuroinflammatory self-maintenance and neural damage (116). The study by Erny et al. (88), showed that germ-free (GF) mice and mice treated with antibiotic therapy exhibited altered microglial responses following LPS inoculation; however, these alterations were partially restored following the recolonization of the intestinal microbiota and the consequent restoration of SCFA production. This study suggests that intestinal eubiosis, also through the correct production of SCFAs, is essential for microglial functional homeostasis. SCFAs can strengthen barrier integrity by regulating tight junctions (occludin, claudin, ZO-1), thus strengthening “enteric immunity” and preventing the translocation of bacteria and potential pathogens. On the contrary, studies conducted in patients with autism spectrum disorder (ASD) show how intestinal dysbiosis favors barrier permeability and the translocation of potential pathogens, contributing to neuroinflammation and worsening of social, cognitive, and behavioral domains (117).

The gut microbiota is also able to communicate with the immune system, “training” it to perform its specific tasks. To facilitate immune surveillance, the intestine is interconnected with the immune cells of the Gut-Associated Lymphoid Tissue (GALT); the epithelial layer is composed of intraepithelial lymphocytes and T cells, while the underlying layer (lamina propria) contains myeloid cells and T cells. The latter include CD4+ regulatory T cells, which are essential for maintaining immune tolerance, preventing autoimmune responses, and controlling inflammation. Other cells that act as sentinels are dendritic cells (DCs), which extend their dendrites toward the intestinal lumen to sample the antigens present. Finally, the luminal side of the epithelial layer expresses numerous intracellular adhesion molecules-1 (ICAM-1), which, following pro-inflammatory stimuli, recruit neutrophils to the luminal side of the colon (118).

This complex immune surveillance system plays an important regulatory role in the central barriers. Both the BBB and the GVB are highly selective, while the PVB is highly permeable, thanks to its fenestration that allows the passage of water and solutes such as glucose and amino acids. This permeability, however, is strongly determined by PV-1 (Plasmalemmal Vesicle-associated protein-1) and is modified by the inflammatory state. PV-1 is a transmembrane protein capable of regulating the closure of fenestrated capillaries, modulating the translocation of immune molecules and cells between the blood and surrounding tissues (66, 67).

Rescigno et al. (66) showed that under conditions of eubiosis, the GVB is perfectly functional and allows nutrients and beneficial bioproducts to enter the bloodstream, blocking the access of bacteria and toxins. In this way, blood flow can be considered “safe” for the CNS, and the PVB can remain open and allow maximum exchange of molecules and immune cells. In a certain sense, it is as if the functional integrity of the intestine guarantees that anything that can reach the systemic circulation can also be considered “safe” for the CNS. Conversely, under conditions of inflammatory intestinal dysbiosis, the protective function of the GVB is compromised and can no longer guarantee the safety of anything that, through the circulation, can reach the nervous tissue. In this condition, the “rescue” function of the PVB is activated, drastically closing the gates to prevent unwanted molecules from reaching the CNS; However, this closure is nonselective and, on the one hand, hinders the entry of molecules essential for the synthesis of neurotransmitters into the CNS. On the other hand, it prevents the removal of catabolic and neurotoxic products from the CNS, effectively compromising neuronal physiology (68, 69).

Finally, the HPA axis is one of the main regulatory factors in response to stress. Indeed, its activation leads to the release of corticotropin (CRH) at the hypothalamus, the production of adrenocorticotropic hormone (ACTH) at the pituitary level, and the production of cortisol at the adrenal cortex. The HPA axis develops early in the prenatal period and continues to mature after birth. Stressful events during this critical window can negatively impact development, impacting cognitive, emotional, and behavioral growth in childhood (119). Addressing these aspects, starting with the gut microbiota as a key to immune modulation and neuroinflammation, lays the foundation for prospects in both scientific research and clinical practice. From the few studies conducted, the literature highlights that children with PANS/PANDAS, compared to their healthy peers, exhibit lower α-diversity, a significant reduction in *Firmicutes* (large butyrate producers), and a notable increase in *Bacteroidetes*, with increased gram-negativity. This imbalance represents the pathophysiological basis for the loss of barrier integrity, increased circulating LPS, and endotoxemia, resulting in worsening neuroinflammation and neuropsychiatric symptoms.

Quagliariello et al., 2018 show that younger PANS/PANDAS patients (4–8 years old), and therefore those least exposed to therapeutic interventions that may have altered the naive microbial signature that characterizes these patients, are characterized by high levels of *Bacteroidaceae* and *Odoribacteriaceae*, while *Tissierellaceae, Gemellaceae*, and *Carnobacteriaceae*, belonging to the *Firmicutes phylum*, and *Bifidobacteriaceae* are absent. However, in the future, it will be necessary to develop randomized, controlled, double-blind studies with a clear identification of well-defined target populations, with systematic collection of metadata such as diet, lifestyle, medication intake, demographic factors, stratifying the study sample based on these key variables. However, future longitudinal and interventional studies will be crucial to clarify causality and define the clinical significance of gut–brain–immune interactions in PANS/PANDAS (120).

## 5 Oral microbiota and its contribution

### 5.1 The oral-gut-brain axis: a novel framework

The human oral cavity is also home to a complex and dynamic microbiota with over 700 bacterial species that play a critical role in maintaining oral homeostasis. Alterations in this microbial balance are associated with oral diseases such as periodontitis (characterized by overgrowth of *Porphyromonas gingivalis, Tannerella forsythia* and *Treponema denticola*), caries (characterized by the predominance of acidogenic and aciduric bacteria such as *Streptococcus mutans* and *Lactobacillus* spp. and oral fungal overgrowth (especially of *Candida albicans (*121), often triggered by antibiotic use or immunosuppression (122).

Recent studies are showing that oral dysbiosis can also support systemic inflammation, thus negatively impacting global health, including mental health (123, 124).

New evidence suggests that oral inflammatory bacteria such as *Fusobacterium nucleatum* and *P. gingivalis*, in addition to having a local inflammatory action, can also perform a distant action. In fact, these oral microorganisms can translocate directly into the gut, altering the microbiota and potentially influencing the appearance of metabolic disorders, and can also support the up-regulation of pro-inflammatory cytokines such as IL-6 and TNF-α that exacerbate systemic inflammation and neuroinflammation by triggering a vicious circle that reverberates on itself (125–127).

**Figure 3:**
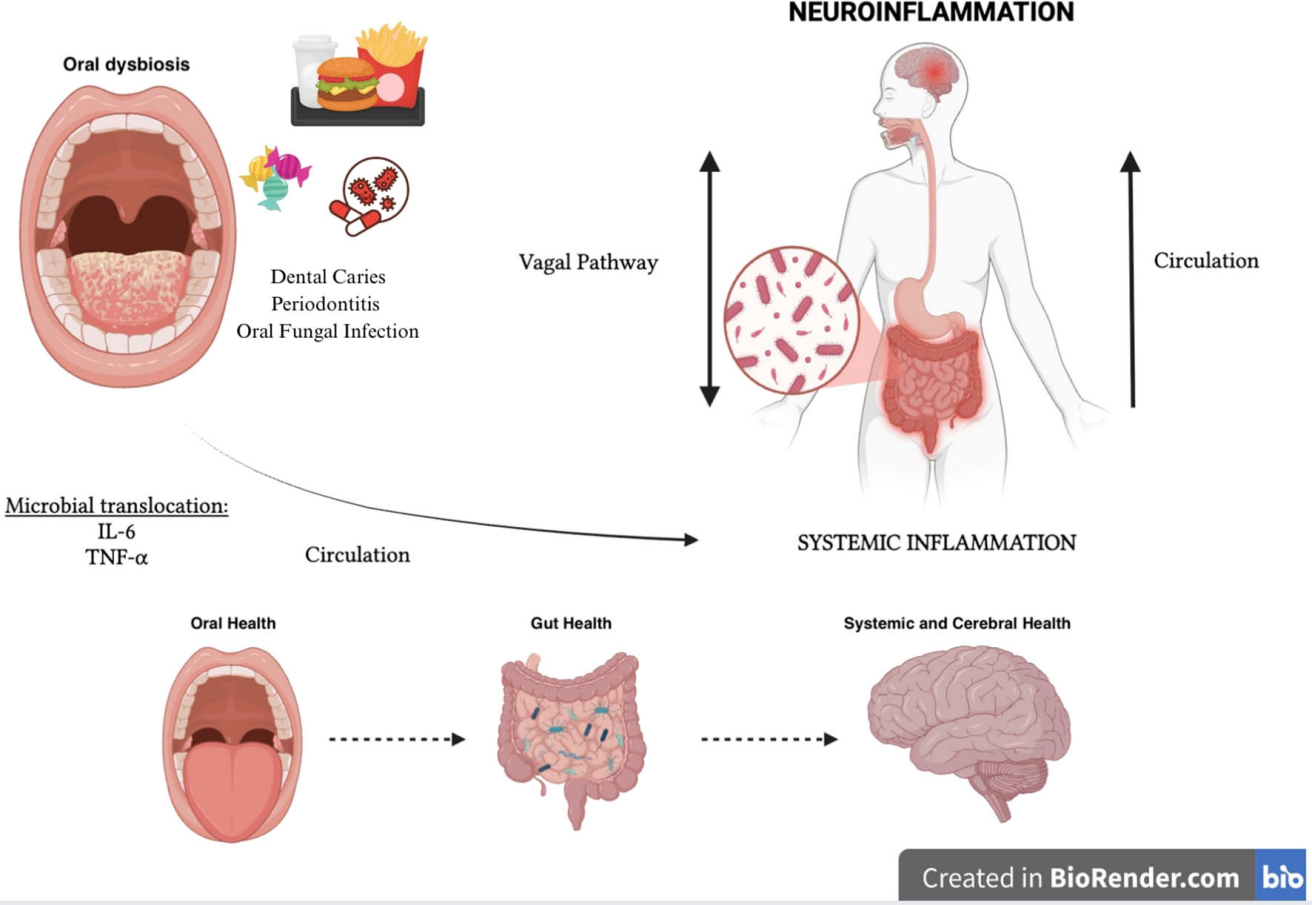
Oral-gut-brain axis.

A new gut-organ axis has thus been identified, the “Oral-Gut-Brain Axis” represented by a bidirectional communication network in which metabolites and immune mediators of oral and intestinal microbial derivation, through the bloodstream and the vagal system, regulate both oral and intestinal and mental health (125, 127, 128).

(**Figure 3**) In fact, it seems that the oral dysbiotic microbial community has evolved to evade the host immune response mediated by neutrophils, which are the leukocytes most recruited in the subgingival sulcus and periodontal pockets, while promoting the inflammatory aspects indispensable to the oral dysbiotic community to obtain, from tissue degradation, nutrients such as degraded collagen peptides and heme-containing compounds. Periodontal bacteria strategically subvert complement activation through the degradation of C3 or key upstream components (such as mannose-binding lectin), to interfere with the neutrophil-mediated destruction action while maintaining a persistent inflammatory environment (123, 129, 130). Additional immunosuppressive mechanisms implemented by oral pathogens include the ability to produce proteases such as gingipain, carilysine, and interpain A (expressed respectively by *P. gingivalis, T. forsythia*, and *P. Intermedia*), capable of inactivating human antimicrobial peptides such as β-defensins. In addition, *P. intermedia* and *T. denticula* can also escape complement-mediated killing through inactivation of factor H (physiological complement inhibitor) (123, 131). *P. gingivalis* has also been shown to manipulate adaptive immune responses by promoting the differentiation and recruitment of CD4 + 17 (Th17) helper T cells at the expense of the Th1 lineage (132).

Oral dysbiosis through the Oral-Gut-Brain Axis also triggers and/or compromises and maintains intestinal dysbiosis and the production of microbial mediators such as SCFAs which, acting on the regulation of the integrity of peripheral and central barriers, on the activation of microglial cells and on neurotransmitter synthesis, can influence both the local oral inflammatory state and the systemic inflammatory state by supporting neuroinflammation and cognitive function. In addition, oxidative stress induced by oral dysbiosis leads to the deterioration of mitochondrial function in both neural and periodontal tissues, and this can further support and exacerbate the inflammatory state (125, 127, 133–135).

In the future, the functional understanding of this new microbiota-organ axis could open new perspectives for therapeutic intervention to counteract neuroinflammation.

## 6 Biomarkers and microbiota-associated inflammation

### 6.1 LPS, NOX2, zonulin, and oxidative stress

Recent evidence documents alterations in the microbiota of children with PANS/PANDAS^105^ and indicates that lipopolysaccharide endotoxin, produced by Gram-negative bacteria, can induce neuroinflammation by increasing oxidative stress. In several pathological conditions, including non-alcoholic fatty liver disease (NAFLD), pneumonia, atherosclerosis, and neurodegenerative diseases, a pathogenetic interaction between LPS, oxidative stress, and activation of the enzyme NADPH oxidase 2 (NOX2) has been described.

Based on these correlations, Loffredo et al. (2020) (136) hypothesized that NOX2 overactivation occurs in subjects with PANDAS, associated with increased oxidative stress, thus contributing to the onset and maintenance of the disease. Gram-negative bacteria from the gastrointestinal tract release LPS with proinflammatory action on neurons. Experimental animal studies have shown that LPS can amplify neuroinflammation by activating NOX2. Starting from this evidence, the authors measured two markers of oxidative stress in the serum of children with PANDAS and healthy controls, sNOX2-dp and 8-iso-prostaglandin F2α (8-iso-PGF2α) and evaluated the role of gut-microbiota-released LPS in inducing systemic NOX2 activation.

The results showed that, in subjects with PANDAS compared to healthy controls:

- Serum levels of sNOX2-dp, 8-iso-PGF2α, LPS, and zonulin were significantly higher.
- LPS was linearly related to both sNOX2-dp and isoprostanes, suggesting a direct correlation between LPS and oxidative stress.
- The increase in sNOX2-dp and isoprostanes indicated an increase in NOX2-mediated systemic oxidative stress.
- The severity of tics correlated positively with isoprostane levels, highlighting an association between oxidative stress and neuropsychiatric symptoms.
- Zonulin levels, an indicator of intestinal permeability, were higher and correlated with serum LPS levels, suggesting increased intestinal permeability that could facilitate the passage of LPS into the bloodstream.

### 6.2 “Th17/IL-17 and regulatory T cells (Tregs)”

_Among cytokine-mediated pathways, particular attention has been drawn to the Th17/IL-17 axis, which plays a pivotal role in linking peripheral immune activation with central neuroinflammation. As discussed in Section 3.2, IL-17 elevation has been detected in serum and CSF of affected children, supporting its potential as a biomarker of neuroimmune activation and a candidate target for therapeutic modulation. Treg cells play the opposite role to Th17 cells in the immune response; in fact, they mediate immune tolerance, representing the key to suppressing the excessive inflammatory activity of Th17 cells. Treg cell differentiation is driven by the forkhead box transcription factor P3 (FoxP3), which is reduced in individuals with inflammatory neurological diseases, along with an upregulation of RORγt.

The imbalance between Th17 and Treg cells is crucial in autoimmunity; however, their role in neurological and mental disorders has not yet been fully elucidated. Certainly, the imbalance of the Th17/Treg ratio correlates with alteration of the expression of microglial phenotypes, so the improvement of the imbalance of the Th17/Treg ratio may be one of the therapeutic targets also in PANS/PANDAS (137, 138).

### 6.3 Secondary bile acids

Emerging data demonstrate the involvement of secondary bile acids produced by the microbiota in the attenuation of neuroinflammatory processes (139). The enzymes produced by the intestinal microbiota can transform primary bile acids, produced in the liver, into secondary bile acids; these (deoxycholic acid, lithocholic acid and ursodeoxycholic acid) are absorbed by enterocytes and mainly reach the liver and in low concentrations the specific receptors ubiquitously expressed in various organs and tissues such as the CNS, in particular BBB, choroid plexus and glial cells (140).

This suggests that bile acids may play a role in regulating glial cell function. In fact, *in vitro* studies have highlighted the beneficial effects of bile acid supplementation on neuroinflammation processes.

Mouse microglial cell lines treated with ursodeoxycholic acid (UDCA) showed a reduction in the expression of TNF-α and reactive oxygen species (ROS) induced by previous LPS treatment (141). Protective effects of UDCA against oxidative stress and neuroinflammation have also been detected in *in vivo* studies of mouse models of Parkinson’s disease (PD) (142). Primary microglial cells, derived from rats exposed to tauroursodeoxycholic acid, showed lower inducible nitric oxide synthase (iNOS) mRNA and increased anti-inflammatory gene expression in response to previous LPS treatment (143).

### 6.4 Tryptophan metabolites

Tryptophan in the diet can be fermented by the microbiota, giving rise to various indole-containing compounds, including indole-3-lactic acid, indole-3-acrylic acid, indole-3-propionic acid, indole-3-acetic acid, indole-3-aldehyde, and other intermediates (144). Recent evidence has demonstrated the involvement of these metabolites in the regulation of functional expression of microglia in an anti-inflammatory M2 sense, in suppressing the production of pro-inflammatory cytokines in astrocytes, and in alleviating neuroinflammation in mice treated with antibiotics. Tryptophan-derived metabolites act as ligands for the aryl AhR receptor (145, 146) (expressed in various cell types such as dendritic cells, microglia, and astrocytes), limiting the production of inflammatory cytokines such as IL-6, TNF-α, and iNOS in astrocytes (146).

### 6.5 Bacterial biomarkers

Several authors agree on the hypothesis that Streptococcus infections can alter bacterial communities by selecting pro-inflammatory bacterial strains and altering the production of neuroactive metabolites (e.g., SCFAs, intermediates of D-alanine metabolism, tyrosine, and dopaminergic pathway), with possible behavioral repercussions in PANS/PANDAS patients. In addition, the selection of proinflammatory strains can support persistent inflammatory immune activation even after the infection has resolved. Further studies have shown that, in PANDAS, clinical exacerbations can be triggered by other infections or stress, both known negative modulators of the microbiota (147).

These data support the interest in specific bacterial biomarkers, potentially useful for developing targeted modulation strategies (dietary interventions or selective probiotics). Approaches already validated in the study of the microbiota-gut-brain axis include germ-free models, antibiotics, microbiota transplantation, and probiotics; for PANS, the development of mouse models integrating vulnerability factors and known triggers, such as alteration of immune function, allergies, respiratory infections, and stress, has been proposed (148).

However, the available clinical studies are preliminary and rarely include essential metadata (maternal health, gestational age at birth, mode of delivery and breastfeeding, early antibiotic exposure, use of psychotropic drugs, diet, lifestyle) indispensable in interpreting the stabilization and modulation of the gut microbiota; therefore, the possibility of obtaining precise microbial signatures in PANS/PANDAS patients is not conclusive at the moment. It would therefore be desirable to carry out case-control studies that evaluate the clinical metadata and their relationship with the characteristics of the oral and intestinal microbiota of patients with PANS/PANDAS at an early stage, at diagnosis, as well as during clinical exacerbations. This would make it possible to identify a microbial signature that really corresponds to the naïve patient, his background, and ultimately to the pathological characteristics before empirical therapeutic interferences (especially for antibiotics use), but also with respect to the impact of microbial/proinflammatory triggers in exacerbations.

Immune dysregulation associated with neuroinflammation represents a mechanism common to multiple neurological and neuropsychiatric conditions (ASD, Alzheimer’s, epilepsy, anxiety, depression, PANS/PANDAS) (44), so the identification of microbiota-mediated biomarkers could have diagnostic, prognostic, and therapeutic relevance, guiding personalized microbial modulation strategies.

However, it should be emphasized that further important obstacles with respect to the use of these biomarkers are: the poor standardization of cut-offs and individual variability related to lifestyle and diet, environmental exposure, and drugs; therefore, new longitudinal studies on statistically significant cohorts of patients will certainly be needed to define their role and clinical application.

Potential non-invasive, serum and fecal biomarkers of neuroinflammation related to intestinal and/or oral dysbiosis are proposed in **Table 5**.

**Table 5:** Serum biomarkers and fecal biomarkers.

Serum biomarkers:
- Proinflammatory cytokines: IL-1β, IL-6, TNF-α, INF-γ, IL-17
- Ratio: Th17/Treg
- LPS
- sNOX2-dp
- 8-iso-prostaglandin F2α (8-iso-PGF2α)
- SCFAs (Acetate, Propionate, Butyrate)
- Indole metabolites of tryptophan (Indole 3 acetic IAA, Indole 3 propionic PAH, indole 3 lactic ILA, indole 3 aldehyde IAID)
- Secondary biliary acids (DCA deoxycholic acid, LCA lithocholic acid, and ursodeoxycholic acid UDCA)
Fecal biomarkers
- Zonulina
- LPS
- Taxonomy and indices of gut microbial α-biodiversity
- SCFAs (Acetate, Propionate, Butyrate)
- Indole metabolites of tryptophan (Indole 3 acetic IAA, Indole 3 propionic PAH, indole 3 lactic ILA, indole 3 aldehyde IAId)
- Secondary bile acids (DCA deoxycholic acid, LCA lithocholic acid and UDCA ursodeoxycholic acid)

## 7 Therapeutic perspectives

Growing evidences demonstrates how microbiota are capable of interacting with the brain through extremely complex, bidirectional communication. Therefore, considering this evidence, the need to investigate the microbial signatures that may characterize specific clinical conditions appears increasingly urgent. Analysis of the gut microbiota could be crucial in understanding and therapeutic/clinical management of PANS/PANDAS. This could not only offer new diagnostic and prognostic tools, identifying specific biomarkers associated with dysbiosis (149), but could also enable the identification of therapeutic interventions to alleviate neuropsychiatric symptoms through lifestyle and dietary modification, as well as through the administration of prebiotics, probiotics, and postbiotics, or fecal microbiota transplantation (FMT) (150).

Quagliarello et al. highlighted how the microbial signature characterizing naive PANS/PANDAS with low alpha diversity, high levels of *Bacteroidaceae* and *Odoribacteriaceae*, and reduced *Bifidobacteriaceae.* However, in the future, it will be necessary to develop randomized, controlled, double-blind studies with clear identification of well-defined target populations. These studies should systematically collect metadata, including diet, lifestyle, medication intake, and demographic factors, and stratify the study sample based on these key variables (120). It is also important to note that in clinical management, these patients are subjected to prolonged antibiotic therapy, which represents a reduction in microbial biodiversity, particularly leading to a decline in *Bifidobacteria*. Jaime Ramirez et al. show how a four-day course of antibiotics can induce significant changes in bacterial biodiversity over a 180-day follow-up period. During this observation period, several species were undetectable, with a particular decline in *Bifidobacteria* (151, 152). These species are important due to their ability to produce acetate (a precursor to butyrate). Their reduction, therefore, leads to a further deterioration in the structure and function of the intestinal barrier, with increased intestinal permeability, resulting in proinflammatory microglia hyperactivation and worsening neuroinflammation.

To date, scientific evidence supports the idea that nutrition and the intake of specific probiotics, prebiotics, and postbiotics represent an important opportunity for modulating the intestinal microbiota. Thanks to the foods consumed, the intestinal microbiota can ferment numerous substances and provide metabolic products such as SCFAs, which are able to establish bonds with free fatty acid receptors (FFARs). Hydrocarboxylic acid receptors (HCARs), G-protein-coupled receptors (GPCRs), fine-tune the secretion of intestinal hormones and neurotransmitters such as PYY, GLP-1, GABA, and 5-HT, regulating energy metabolism, barrier integrity, and the morphology and function of microglia in a pro- or anti-inflammatory manner (133).

Moreover, considering recent scientific evidence, studying the fecal microbiota could open the possibility of developing personalized nutraceutical therapy to support the health of these young patients through the use of prebiotics, probiotics, and postbiotics.

### 7.1 Prebiotics, probiotics, postbiotics

#### 7.1.1 Prebiotics

Prebiotics are defined by the ISAPP (International Scientific Association for Probiotics and Prebiotics) as “a substrate that is selectively utilized by host microorganisms, conferring a health benefit.” These include certain oligosaccharides such as FOS (fructooligosaccharides) and GOS (galacto-oligosaccharides), which are not digested by humans but promote the growth of *Bifidobacteria* and *Lactobacilli.* GOS, derived from lactose, consists of a glucose molecule linked to one or more galactose units and is selectively fermented by *Bifidobacterium* spp. and *lactobacilli*, thus playing an important bifidogenic role. Therefore, FOS, which includes inulin, XOS (xylo-oligosaccharides), and IMO (isomaltose-oligosaccharides), is our soluble fiber found in chicory, Jerusalem artichoke, garlic, onion, and unripe bananas, and is selectively fermented by *Bifidobacteria.* 143 Furthermore, inulin obtained from chicory roots has a prebiotic effect on bifidobacteria and lactobacilli, and on *F. prausnitzii*, a large butyrate-producing bacterium.

The common role of prebiotics is therefore to stimulate the growth of SCFA-producing bacteria, which strengthen the integrity of the intestinal barrier, exhibit immunomodulatory effects, and reduce systemic inflammation. A study by Chunchai et al. (153) showed that obese mouse models with insulin resistance, when supported with a prebiotic (xylooligosaccharide), probiotic, or both (synbiotic), showed reduced intestinal and systemic inflammation, improved hippocampal plasticity and mitochondrial function, and decreased microglial activation and oxidative stress/brain apoptosis (153). These findings highlight a shared mechanistic pathway in which SCFA restoration and microglial modulation converge to maintain neuroimmune balance. Recent evidence indicates that SCFAs directly modulate microglial activity through several molecular pathways. In particular, butyrate and propionate can cross the BBB and act via G-protein–coupled receptors (GPR41, GPR43, GPR109A) expressed on microglia, leading to suppression of proinflammatory signaling (e.g., NF-κB and MAPK pathways). Moreover, SCFAs function as histone deacetylase (HDAC) inhibitors, promoting an anti-inflammatory and neuroprotective transcriptional profile characterized by increased expression of IL-10 and neurotrophic factors. Through these epigenetic and receptor-mediated mechanisms, SCFAs help restore microglial homeostasis, limit oxidative stress, and support synaptic integrity within the CNS (154).

Among prebiotics, Human Milk Oligosaccharides (HMOs) have attracted great interest; a class of complex carbohydrates abundantly present in human breast milk, representing the third most abundant solid component after lactose and lipids, with an average concentration of approximately 11 g/L in mature milk (155). Currently, over 200 different molecular structures of HMOs have been identified. HMOs are indigestible by human intestinal enzymes and therefore reach the colon unchanged, where they are selectively fermented by beneficial bacteria, particularly *Bifidobacterium* species such as *B. infantis, B. bifidum, B. breve*, and *B. longum*. This fermentation produces SCFAs through the activation of specific cross-feeding mechanisms.

Recently, it has been shown that infant-type *bifidobacteria (B. longum, B. infantis, B. bifidum*, and *B. breve*) are also among the few probiotic microorganisms with aldehyde dehydrogenase (ALDH), capable of metabolizing aromatic amino acids such as tryptophan, phenylalanine, and tyrosine into aromatic lactic acids such as indol-lactic acid (ILA), phenylalactic acid (PLA), and 4-hydroxyphenyllactic acid (4OH-PLA). Aromatic lactic acids act on the aryl receptors AhR and hydroxycarboxylic acid HCA3 expressed on cells of the innate immune system, promoting the release of IL-22, which is implicated in promoting stem cell differentiation and the integrity of the intestinal barrier and BBB. They also inhibit the secretion of IL-12p70, a highly inflammatory cytokine (156).

Furthermore, HMOs perform immunomodulatory functions as they can act as decoy receptors, preventing pathogens from adhering to intestinal epithelial cells, and can modulate the immune response by binding to transmembrane Toll-like receptors (TLRs). HMOs are also able to modulate extravascular migration, as by bind to endothelial lectins (proteins that play a crucial role in cell-cell adhesion), preventing activated leukocyte cells from adhering and migrating from the blood into tissues (157). Observational studies have associated early exposure to specific HMOs, such as 2′fucosyllactose (2′FL) and 6′-sialyllactose (6′SL), with improvements in cognitive, linguistic, and motor development in the first two years of life (158). Moreover, an Italian observational study conducted on 52 children diagnosed with PANS showed that exclusive breastfeeding, a natural source of HMOs, is associated with better neurodevelopmental outcomes. Growth problems were not observed in breastfed children (0% *vs*. 12.9% in non-breastfed children), the onset of language was earlier (4–20 months *vs*. 7–36 months), and the need for school support was lower (19.1% *vs*. 29%). These data suggest a potential protective role of HMOs in the neurodevelopment of children with PANS, through modulation of the microbiota and reduction of neuroinflammation.

#### 7.1.2 Probiotics

Probiotics are defined by the ISAPP as “live microorganisms that, when administered in adequate amounts, confer a health benefit on the host”. This definition emphasizes that probiotics must be alive, given in sufficient quantities, and supported by evidence demonstrating a measurable health effect. One precision bacterial strain is *Bifidobacterium breve* PRL2020, which shows intrinsic and nontransferable resistance to amoxicillin, amoxicillin-clavulanate, and ampicillin, thus capable of supporting the colonic microbiota even in combination with that antibiotic therapy (159). Moreover, *C. butyricum* CBM588, a spore-forming strain, has proven highly effective during antibiotic therapy. It sporulates during antibiotic therapy and, when the antibiotic is stopped, emerges and colonizes. Being a butyrate producer, it promotes anti-inflammatory activity in the intestine and microglia, making it extremely promising in the treatment of depression, anxiety, ASD, and PANS/PANDAS (160). Although butyrate’s epigenetic effects are well-documented in animal models of neuroinflammation, caution is warranted when extrapolating these findings to humans. Differences in physiology, immune responses, and microbiota composition may affect the relevance of animal data. Therefore, careful interpretation is needed, and future studies should aim to validate these effects in human neuro-inflammatory conditions.

*Bifidobacterium adolescentis* PRL2019, a major GABA producer, could be another promising probiotic and may play a role in the control of tics, anxiety, depression, sleep disorders, pain modulation, and PANS/PANDAS (161, 162). Given the known trigger role of upper respiratory tract infections in triggering/reactivating PANS/PANDAS, *Streptococcus Salivarius BLISK12* could have clinical application, both for the antibacterial effect of salivaricin A and B, which act as metabolic poisons against the main upper respiratory tract pathogens (*Streptococcus pyogenes, Streptococcus pneumoniae, Haemophilus influenzae*, and *Morexella catarrhalis*), and for its demonstrated anti-inflammatory effect by reducing the NF-κB cascade, thus preventing the transcription and translation of pro-inflammatory factors such as TNF-α, IL-6, and IL-8 (163).

Furthermore, an engineered strain of BLISK12, called eK12SAL, has recently been released onto the European market. In addition to the well-known salivaricins A2 and B, it produces a third batteriocin called Salivabactin. It is a polyketone/peptide hybrid (pK/NRP) that forms a mixture of two geometric isomers (salivabactin A and B) with extraordinary potency against *Streptococcus pyogenes, Streptococcus pneumoniae, Streptococcus agalactiae*, and *Staphylococcus aureus*, with an MIC of 2 micrograms/ml comparable to that of penicillin G. This strain has been tested in adults (study NCT06380270) and has been shown to eliminate *Streptococcus pyogenes* from human saliva and drastically reduce its presence in the nasopharynx. Furthermore, it was found that eK12SAL significantly reduces IL-1β (a known inflammatory marker associated with pyogenes infection).

A randomized, double-bind, parallel prophylactic pediatric trial (NCT06370208) is currently underway in children aged 3–10 years with a history of recurrent pharyngitis, testing the prophylactic efficacy of eK12SAL. The study will be concluded at the end of the year, and we are awaiting the results (164, 165).

Furthermore, *Streptococcus Salivarius* M18 has also shown itself to be extremely promising and safe, thanks to the proven absence of virulence factors or transferable antibiotic resistance, in combating oral dyskinesia associated with systemic inflammation and neuroinflammation. A study conducted by Salim and colleagues showed that in a cohort aged 3–6 years, a 7-day administration led to a statistically significant reduction in *S. mutans*, which is one of the key bacteria in oral inflammation and dysbiosis and the development of dental caries (166).

A recent 2025 trial in patients with stage III-IV periodontitis, oral administration of M18, as an adjuvant to non-surgical periodontal therapy, showed significant improvements in gingival pocket depth (PPD), plaque index (PI), and bleeding (BoP) compared to a placebo, with consequent improvement in oral eubiosis and reduction of pathogens. *Streptococcus Salivarius* M18, in addition to producing bacteriocins capable of counteracting competing pathogens, is also capable of producing enzymes such as urease and dextranase that enhance its activity against *S. mutans*. It therefore appears to be extremely promising in modulating the oral microbiota, shifting the balance toward an eubiotic and less pathogenic profile (167). Although these probiotic strains have demonstrated a safety profile in both preclinical and clinical studies, safety considerations remain crucial, particularly in the pediatric population. However, the developing gut microbiota and immature immune system may influence host–microbe interactions and susceptibility to adverse effects. Although rare, cases of probiotic-associated bacteremia, fungemia, or translocation have been described, primarily in immunocompromised, preterm, or critically ill children. Therefore, strain-specific safety data and rigorous quality control of probiotic formulations are essential. The use of well-characterized strains with documented safety and absence of transferable antibiotic resistance is strongly recommended. In clinical practice, careful monitoring and individualized assessment should guide probiotic administration in pediatric patients, especially when concomitant antibiotic or immunosuppressive therapy is involved (168–173).

Recent clinical and technological evidence has deepened the understanding of the potential effects of probiotics as microbiome-based interventions, exploring the underlying immuno-inflammatory and metabolic mechanisms. A randomized controlled trial (NCT02957591) demonstrated that four weeks of probiotic supplementation resulted in a significant reduction in depressive symptoms, accompanied by an increase in the gut hormone ghrelin and an upregulation of immune-related genes, including ELANE, DEFA4, and OLFM4, suggesting a role in neuroimmune modulation (174, 175).

In parallel, advances in synthetic biology have enabled the development of engineered probiotics designed to act as diagnostic or therapeutic tools across a range of diseases — from gastrointestinal and metabolic disorders to neoplastic and infectious diseases — opening new avenues for targeted modulation of the microbiome and immune activity (176).

Overall, converging evidence suggests that modulation of the gut microbiota, for instance through probiotic supplementation – whether traditional or engineered – may influence neuroimmune and metabolic physiology, representing a promising integrated approach to the management of multifactorial conditions.

#### 7.1.3 Postbiotics

The microbiome therapeutic approach also makes use of postbiotics, for which two very different definitions currently coexist. ISAPP has defined them as biologically active substances derived from microorganisms (especially bacteria) or their components, which confer health benefits to the host, without the need for the microorganisms to be viable (177). Meanwhile, the authoritative journal Nature has published a further definition that sees postbiotics as a standardized mixture of stable and reproducible microbial metabolites in the absence of other structural components (178).

One example of a postbiotic among the most studied and documented to date is the amuc 1100 wall component of *Akkermansia muciniphila*, a strain extremely active in mucus production, the regulation of tight junctions (TJs), the downregulation of pro-inflammatory cytokines, and the promotion of neurogenesis by protecting against oxidative stress. It could therefore also have a promising role in PANS/PANDAS patients (179).

### 7.2 Diet and microbiota modulation

Finally, in addition to targeted microbiota approaches through individualized supplementation, nutrition plays the role of the greatest modulator of the intestinal microbiota, whose signature responds in a highly individual manner based on daily food choices, inducing specific changes in the diversity and abundance of different microbial species, with effects measurable through host metabolic and inflammatory parameters (180). As shown by Toshchakov et al. (2018), even brief personalized dietary interventions can produce profound changes in the structure of the microbial community, and the response depends on the initial state of everyone’s microbiota (181). Elinav and colleagues (2019) highlight that the integration of clinical metadata and the host microbiota allows for the development of precision nutritional strategies, with the aim of improving health and preventing various chronic diseases (182).

#### 7.2.1 Fermented foods

Among the most promising strategies, current scientific evidence suggests the regular inclusion of fermented foods such as yogurt, kefir, kimchi, kombucha, miso, tempeh, fermented vegetables and legumes, and cheeses. Fermented foods are food products obtained through microbial fermentation (lactic, acetic, propionic, or alcoholic) of raw food materials such as dairy products, vegetables, or grains. This process produces a finished food product with high concentrations of probiotics (an effective strategy for enriching microbial biodiversity) and bioactive and organoleptic properties.

Specific microorganisms such as *Bifidobacteria* and *Lactobacilli, Lactococci, Pediococci, Leuconostoc* spp.*, Streptococcus thermophilus*, and *Acetobacter* spp. are used during fermentation. As well as yeasts and molds that produce organic acids (acetic, lactic, butyric, and propionic acids), bioactive peptides (ACE, PPI, VPP, bacteriocins, and antimicrobial peptides), vitamins and transformed polyphenols (aglycones and catechins), and exopolysaccharides (acetans, dextrans, levans, β-glucans, xanthans, and kefir). Fermented foods can therefore contain a wealth of probiotics and postbiotics at the time of consumption (183–185).

#### 7.2.2 Mediterranean diet

To date, based on current evidence, we can affirm that the Mediterranean diet, the gold standard for human health, is also a winning strategy for the health of the intestinal microbiota. It is rich in fruits, vegetables, whole grains, legumes, fish, extra virgin olive oil, nuts, and low in red meat, ultra-processed foods, and refined sugars. The high levels of polyphenols, monounsaturated fatty acids (MUFAs), and omega-3 contribute to the reduction of oxidative stress and chronic inflammation. It also promotes bacteria such as *Faecalibacterium prausnitzii*, a key butyrate producer (186).

#### 7.2.3 Restrictive diets

Based on this evidence, it is now appropriate to discourage the initiation of restrictive dietary protocols in the absence of clear and documented clinical indications of allergy or intolerance. Gluten- and lactose-free diets, often used empirically, are currently not supported by any scientific evidence for improving intestinal biodiversity or for improving clinical conditions such as neurodevelopmental disorders and PANS/PANDAS. In addition to not promoting these clinical conditions, they can represent the beginning of malnutrition due to micronutrient deficiencies, which, if not adequately recognized and supplemented, can contribute to worsening the clinical condition.

Currently, the gluten-free (GF) diet is recommended by ESPGHAN (European Society of Pediatric Gastroenterology, Hepatology and Nutrition) guidelines only for those with celiac disease (187). Furthermore, starting this nutritional regimen can expose patients to a greater consumption of packaged and processed foods and the risk of developing serious deficiencies of folate (B9), thiamine (B1), niacin (B3), and vitamin B12, which are reduced in industrial GF products that use unfortified refined flours (129). Dairy-free diets, meanwhile, can lead to calcium and vitamin D deficiencies (188). Moreover, up to 80% of child-targeted GF products have high sugar content, and nearly all exceed recommended thresholds for sugar, fat, or sodium. Ninety-eight percent of GF products exceed nutrients of concern for fat, sugars, and/or sodium according to PAHO (Pan American Health Organization) criteria. Elliott, C (2025). Assessing the nutritional quality of gluten-free packaged foods for children. *British Food Journal* (189). For these deficiencies, the goal should not be to perpetuate the consumption of fortified UPFs, which remain (although fortified with vitamins and minerals) rich in sugars, additives and vegetable oils, but to promote the consumption of pseudocereals, and foods naturally GF but rich in vitamin B12 such as offal (beef liver), lamb, poultry, fish such as mackerel, salmon, tuna and sardines, eggs and finally dairy products, especially mature ones such as parmesan and pecorino (190).

It should therefore be reiterated that the low nutritional quality of packaged GF products, lactose-free foods, and this evidence is greater for casein-free milks such as vegetable milks, which often contain a greater abundance of sugars to improve their palatability, and restrictive eating habits are in no way justified by current scientific evidence for neurodevelopmental disorders.

### 7.3 Evidence on microbiota-targeted interventions and current gaps

To date, no published or registered clinical trials have specifically evaluated the use of probiotics, prebiotics, or fecal microbiota transplantation (FMT) in pediatric patients with PANS or PANDAS. Available evidence is limited to observational studies, case series, and microbiome–disease association reports, whereas interventional trials have been conducted in other neurological or neuroimmunological contexts (e.g., autism spectrum disorder, depression, multiple sclerosis, Parkinson’s disease) but not yet in the PANS/PANDAS field (150).

The absence of PANS/PANDAS-specific trials may be explained by several challenges: the relative rarity and phenotypic heterogeneity of PANS/PANDAS, ethical and safety concerns in applying FMT in pediatric populations, lack of validated biomarkers for patient selection, and absence of standardized protocols for microbial intervention product preparation and administration (150).

In related neurological disease fields, FMT has begun to be tested in controlled settings. A randomized clinical trial in Parkinson’s disease reported that FMT was generally safe—causing mostly transient gastrointestinal adverse events—but did not demonstrate superior symptomatic benefit over placebo at 6 or 12 months, though microbiome changes were donor-dependent (191).

Other reviews highlight the preliminary nature of FMT in neurology, with human case reports and small trials in disorders like autism, multiple sclerosis and epilepsy, but no definitive evidence yet (150).

Additional controlled trials in Parkinson’s disease (192) further underscore both the promise and limitations of FMT in neurological contexts.

Given this landscape, we propose that future microbiota-targeted research in PANS/PANDAS should proceed methodically: (i) initiate observational and mechanistic “proof-of-concept” studies with immunologic and multi-omic endpoints; (ii) design pilot randomized, controlled trials focusing first on safety and tolerability, before scaling to efficacy; (iii) adopt rigorous donor screening and standardized processing for FMT if used; (iv) utilize multimodal biomarker panels combining metagenomic, metabolomic, and immunological data for stratification and response assessment. Such a stepwise translational approach would help bridge the gap between microbiome observations and safe, effective interventions in PANS/PANDAS.

## 8 Conclusions and future directions

Emerging evidence increasingly supports the hypothesis that alterations of the gut and oral microbiota may contribute to the immune dysregulation and neuroinflammatory mechanisms underlying PANS and PANDAS. Microbiota-derived metabolites and immune mediators appear to modulate the permeability of peripheral and central barriers, microglial activation, and neurotransmitter homeostasis, influencing the expression and progression of neuropsychiatric symptoms. Although current data are still preliminary and heterogeneous, these findings open new perspectives in the understanding of disease pathophysiology and highlight the potential of microbiota-targeted approaches for diagnostic and therapeutic purposes in pediatric neuroimmune disorders (**Table 6**).

**Table 6:** Future directions.

5 Future directions	
Scope	Objective
Prospective studies	Launch multicenter, longitudinal and controlled trials with rigorous methodologies.
Built-in metadata	Correlate clinical, immunological, neuropsychological, and microbiomic data.
Biomarkers	Identify and validate diagnostic and prognostic markers useful in clinical practice.
Personalized therapies	Define protocols based on targeted modulation of the microbiota (diet, probiotics/postbiotics, nutraceuticals, fecal transplantation).
Cooperation	Building interdisciplinary networks between pediatricians, neuropsychiatrists, microbiologists, nutritionists and immunologists.
Prevention	To develop early strategies in at-risk children to reduce the onset of syndromes.

### 8.1 Knowledge gaps and limitations

Despite the growing number of studies exploring the microbiota–immune–brain relationship in PANS/PANDAS, important knowledge gaps remain. Most of the available research is based on small and heterogeneous cohorts, often cross-sectional, with limited statistical power and non-standardized analytical methodologies. In many cases, microbiota profiling has been performed without integrating essential metadata — such as maternal health, gestational age, mode of delivery, breastfeeding, antibiotic exposure, diet, lifestyle, and use of psychotropic or immunomodulatory drugs — all of which can profoundly influence the microbial ecosystem and immune responses. Moreover, longitudinal data comparing disease onset, remission, and exacerbation are lacking, as are studies investigating the oral microbiota and its interactions with the gut–brain axis, despite emerging evidence suggesting that oral dysbiosis may sustain systemic inflammation and neuroimmune activation. Finally, no validated microbial or metabolomic biomarkers are currently available for diagnosis, prognosis, or therapeutic monitoring. These limitations highlight the urgent need for standardized, reproducible methodologies and multicenter collaborative studies designed to clarify causal mechanisms and validate potential biomarkers of disease activity and therapeutic response.

### 8.2 Future research and translational perspectives

Despite the literature reporting few data on this topic, an interesting recent article describes three cases of PANS that improved after treatment with diet, herbal antimicrobials (*Mimosa pudica*, *Triphala*), probiotics (*Saccharomyces boulardii* in one case), and prebiotics (curcumin, cod liver oil/omega-3, resveratrol) (193). Nutritional management, as already mentioned, becomes a fundamental component in the care of these patients due to its ability to interact with their microbiota through dietary prebiotics, while eliminating habits potentially linked to dysbiosis and subsequent neuroinflammation - for example, the consumption of processed foods (194). Regarding the application of fecal microbiota transplantation (FMT), there are reports of two PANS patients who, after antibiotic therapy and FMT for *Clostridioides difficile* colitis, subsequently experienced improvements in other comorbidities, including anxiety, depression, OCD, and rheumatoid arthritis (195).

However, future research should aim to integrate microbiome science with neuroimmunology and clinical pediatrics through multidisciplinary, multi-omic, and longitudinal approaches. Comprehensive analyses combining metagenomics, metabolomics, transcriptomics, and immunophenotyping could help identify specific microbial and metabolic signatures associated with disease phenotypes, clinical severity, and treatment outcomes. Experimental models — including germ-free or humanized mice — may elucidate mechanistic pathways linking dysbiosis, immune activation, and neurobehavioral changes. From a translational standpoint, microbiota-targeted strategies such as personalized nutrition, prebiotics, probiotics, postbiotics, synbiotics, and fecal microbiota transplantation warrant systematic evaluation through well-designed randomized controlled trials with clearly defined endpoints. Equally important will be the development of integrated biomarker panels combining microbial, immunological, and clinical data to support precision diagnostics and personalized interventions. In this context, the integration of microbiomics into pediatric neuroimmunology may contribute to a paradigm shift — from symptomatic management toward preventive and personalized strategies capable of restoring immune–microbial homeostasis and mitigating long-term neuropsychiatric sequelae in affected children.
